# Toothache Remedy

**Published:** 1884-05

**Authors:** 


### TOOTHACHE REMEDY.

"It is claimed that a mild galvanic current, generated by placing a piece of silver (any small silver coin will do) on one side of the gum adjacent to an aching tooth, and a piece of zinc on the other side, and bringing the edges into contact, will immediately stop the pain."—*Medical Exchange.*

The code of Medical Ethics pointedly forbids the recommending or employing, upon the part of reputable physicians, of specific remedies and gimcrack appliances. How is it, then, that we so constantly meet with such silly, unprofessional recommendations as this in medical journals, whose editors would be seriously offended if we accused them of that of which they are really guilty—the most offensive quackery? Is it that they are so culpably ignorant of the plainest principles of dental medicine?

